# Supplementary material for: Application of problem-based learning and case-based learning integrated method in the teaching of maxillary sinus floor augmentation in implant dentistry
Source: PeerJ. 2020 Jan 16;8:e8353. doi: 10.7717/peerj.8353 (PMC6970006; doi:10.7717/peerj.8353)

***Case Analysis***

A 50-year-old female patient wants to have her missing teeth restored by implant supported restoration. Two years ago, she had her left posterior teeth removed due to endodontic failure and severe root fracture. Since then she has to chew with the right teeth and have never worn a fixed or removable restoration. Intraoral examination shows that #26 tooth and #27 tooth are lost. The alveolar ridge is not seriously atrophy and the width of keratinized mucosa is enough. No redness and ulceration of mucosa are observed. #25 teeth has no crown defect and is not inclined to the distal area. The restoration space for #26 and #27 is 7.5 mm, and no obvious elongation of the combined teeth is observed. The mouth opening is normal, and no signs of temporal-mandibular disorder are detected. The condition of oral hygiene is poor, with tartar ++. The patient reports no systemic diseases such as hypertension, diabetes, heart disease, no history of allergies, and no history of taking drugs contraindicated for dental implant placement.

*Answer the following 7 questions using information in the case. Please note that the questions are to be answered one by one in order. The answer for one question must be submitted before you can go to the next one. You cannot retrieve your answer after submission.*

***Question 1***: What tests and examinations are needed to determine whethter the patient can have dental implants or not? (10 points)

***Question 2***: The following pictures are the coronal section of the patient's CBCT at tooth #26 and tooth #27. What anatomical structures should be considered for CBCT measurements? How to measure the key structures? (10 points)


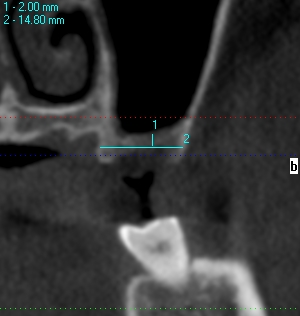

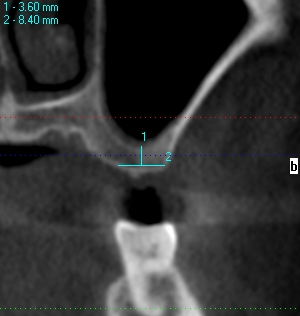


**#27**

**#26**

***Question 3***: According to CBCT measurement, distance between the sinus floor and the crest is 3.6 mm at tooth #26, and 2mm at tooth #27. The width of the alveolar ridge is 8.5 mm at tooth #26, and 15mm at tooth #27. CBCT shows no thickening and inflammation in the mucosa, and no abnormal filling in the maxillary sinus. The floor of the sinus is flat and smooth. No maxillary sinus septum is observed. The thickness of the lateral wall of the maxillary sinus is 1mm-1.5mm, and no blood vessels are found in or near the bony wall. What kinds of restorations can the patient have? If the patient chooses implant-supported restorations, can the patient have dental implants directly? (5 pionts)

***Question 4***: Bone augmentation is indispensable if dental implants are to be placed. What kind of bone augmentation technique is indicated for this patient? Please list your reasons. (5 points)

***Question 5***: According to the patient's condition, we decide to perform maxillary sinus augmentation though lateral wall widow. Can we place the dental implants simultaneously with bone augmentation? Or should we wait until the bone healing is completed? Please list your reasons. (10 points)

***Question 6***: According to the patient's condition, we decide to perform maxillary sinus augmentation though lateral wall widow with simultaneous implant placement. The following pictures are taken during the surgery. Please indicate which step each image represents and list the details and precautions for each step. (40 marks)


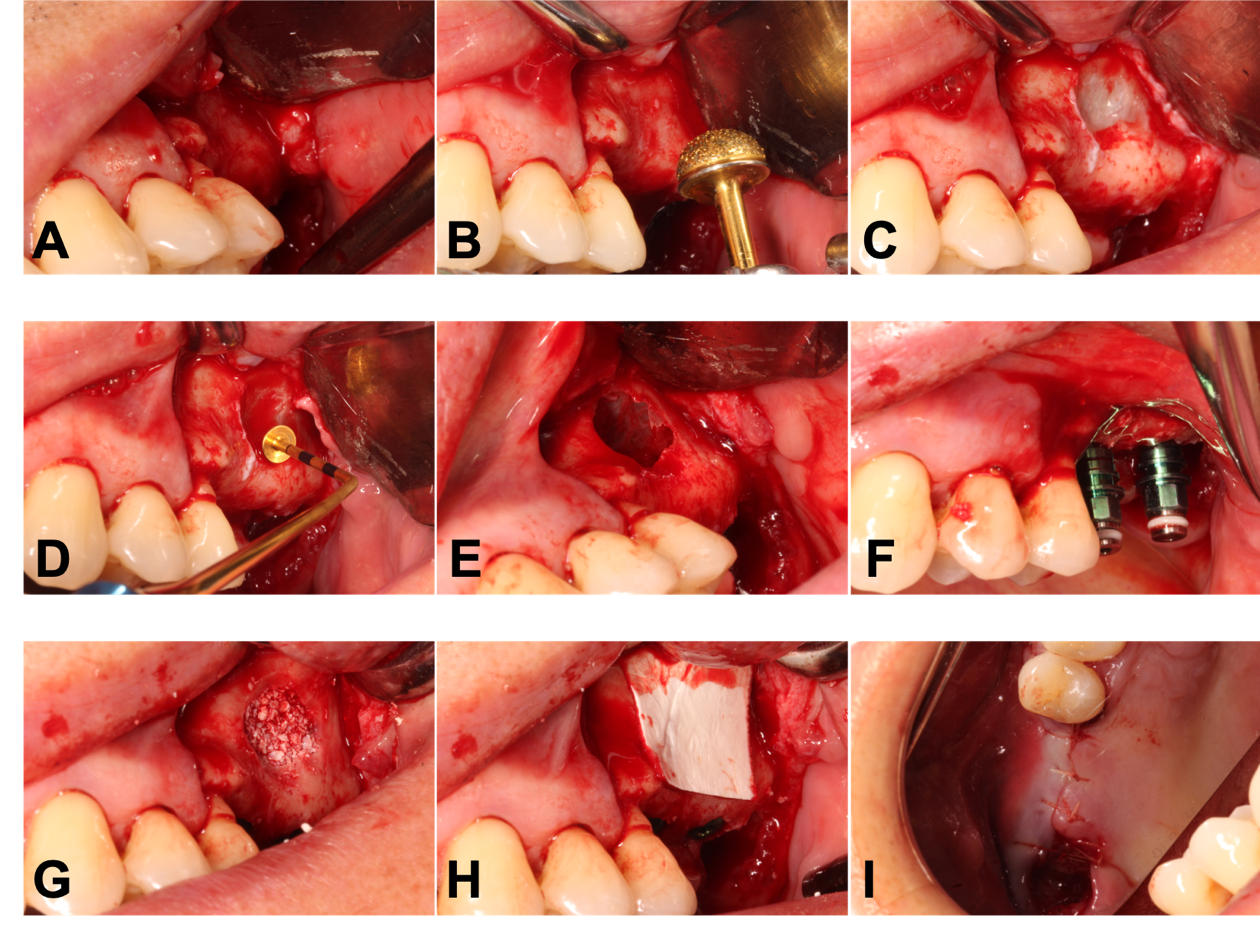


***Question 7***: The postoperative CBCT are shown in the following pictures. Please evaluate the results and list the precautions for postoperative care. (20 points)

**#26**

**#27**


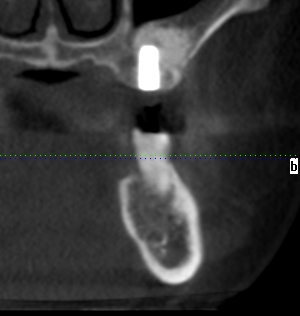

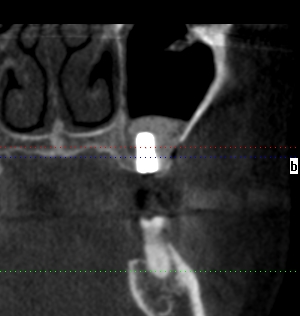

Supplement: Supplemental Information 2 [file peerj-08-8353-s002.docx]
